# CRISPR Cpf1 proteins: structure, function and implications for genome editing

**DOI:** 10.1186/s13578-019-0298-7

**Published:** 2019-05-09

**Authors:** Fatemeh Safari, Khadijeh Zare, Manica Negahdaripour, Mazyar Barekati-Mowahed, Younes Ghasemi

**Affiliations:** 1 Department of Medical Biotechnology, School of Advanced Medical Sciences and Technologies, Shiraz University of Medical Sciences, Shiraz, Iran; 2 Department of Basic Sciences, Faculty of Veterinary Medicine, Ferdowsi University of Mashhad, Mashhad, Iran; 3 Pharmaceutical Sciences Research Center, School of Pharmacy, Shiraz University of Medical Sciences, Shiraz, Iran; 4 Department of Physiology & Biophysics, School of Medicine, Case Western Reserve University, Ohio, USA; 5 Department of Pharmaceutical Biotechnology, School of Pharmacy, Shiraz University of Medical Sciences, Shiraz, Iran

**Keywords:** CRISPR/Cas9, CRISPR/Cpf1, Transcription, Epigenetic, Gene editing, Base editing

## Abstract

CRISPR and CRISPR-associated (Cas) protein, as components of microbial adaptive immune system, allows biologists to edit genomic DNA in a precise and specific way. CRISPR-Cas systems are classified into two main classes and six types. Cpf1 is a putative type V (class II) CRISPR effector, which can be programmed with a CRISPR RNA to bind and cleave complementary DNA targets. Cpf1 has recently emerged as an alternative for Cas9, due to its distinct features such as the ability to target T-rich motifs, no need for trans-activating crRNA, inducing a staggered double-strand break and potential for both RNA processing and DNA nuclease activity. In this review, we attempt to discuss the evolutionary origins, basic architectures, and molecular mechanisms of Cpf1 family proteins, as well as crRNA designing and delivery strategies. We will also describe the novel Cpf1 variants, which have broadened the versatility and feasibility of this system in genome editing, transcription regulation, epigenetic modulation, and base editing. Finally, we will be reviewing the recent studies on utilization of Cpf1as a molecular tool for genome editing.

## Background

Clustered regularly interspaced palindromic repeats (CRISPR) loci are genomic elements that form an adaptive immune system in bacteria and archaea in combination with Cas (CRISPR associated) proteins [1]. The arms race between bacteria and phages has driven the evolution of CRISPR–Cas systems, resulting in six primary types of CRISPR–Cas systems. Types I, III, and IV are defined by multi-subunit effector complexes, while types II, V, and VI are deemed as a single-subunit effector [2, 3].

CRISPR–Cas systems exert their immunity through three distinct stages. In the first stage, called adaptation, the CRISPR system mediates the recognition and acquisition of short DNA fragments from invading viruses and plasmids. These DNA fragments (spacers) are processed and integrated into the CRISPR locus [4]. During the expression as the second stage, the transcription of CRISPR locus to a long pre-CRISPR RNA (pre-crRNA) and the maturation of pre-crRNA to crRNA also termed guide RNA take place [5]. Interference is the last stage in which Cas effector nucleases use the guide RNA to recognize complementary target DNA sequences by Watson–Crick base pairing. After the target DNA recognition, Cas effectors bind to the target DNA and generate a double-stranded DNA break (DSB) [6].

In 2013, two different groups published their findings, which stated the capability of CRISPR to be exploited for genome engineering in mammalian cells [7, 8]. The type II CRISPR/Cas9 system from *Streptococcus pyogenes* (spCas9) is an emerging genome editing tool with broad applications due to its efficiency, easy handling, and simplicity [6]. The signature Cas9 effector proteins are large multi-domain RNA-dependent endonucleases that locate, bind, and cleave the double-stranded DNA (dsDNA) targets which are complementary to their guide RNAs [9].

For recognition and binding to target DNA, Cas9 requires the protospacer adjacent motif (PAM), as a short conserved sequence located just downstream of the non-complementary strand of the target dsDNA [10]. Recognition of the PAM (5′NGG3′) triggers dsDNA melting, enabling crRNA strand invasion and base pairing. The dsDNA cleavage mediation happens via the activity of separate HNH and RuvC nuclease domains. Also, Cas9 is a member of a small subset of Cas effectors that need a second trans-acting crRNA (tracrRNA) for gRNA processing and DNA cleavage [11].

Recently, Zhang and his group at MIT and Broad Institute (USA) discovered a new generation of CRISPR nucleases termed as Cpf1 (CRISPR from *Prevotella and Francisella1*) or Cas12a. This monomeric protein with 1200–1500 amino acids length belongs to type V CRISPR system. Cpf1 CRISPR array consists of nine spacer sequences, which are disassociated by 36 nucleotide long repeated sequences. Cpf1 recognizes a 5′-TTTV-3′ PAM in a DNA target, which leads to the base pairing of the spacer-derived segment of the crRNA with the complementary target DNA. Since Cpf1 simultaneously possesses RNAase and DNAase activity, it does not employ tracrRNAs for crRNA biogenesis; instead, the pre-crRNA forms a pseudoknot, that is recognized and cleaved by Cpf1 itself.

Furthermore, Cpf1 induces staggered ends (5 or 8 nucleotides 5′overhang concerning crRNA length) at the cleaved sites [12]. These unique features introduce Cpf1 as an emerging genome editing tool, which can be used in various biological approaches including multiplex gene targeting, transcription, and epigenetic modulation and base editing [13]. Moreover, the versatility of Cpf1 has led to the usage of this promising tool in the modification of various organisms from prokaryotes to Homo sapiens.

In this review, we will attempt to elaborate on the structural features of Cpf1 and the way these features affect guide RNA binding and processing, DNA targeting, and cleavage. We clarify the basic knowledge that resulted in the engineering of different variants of Cpf1. Subsequently, we will describe the capabilities of DNAase deactivated Cpf1 (ddCpf1) for application in gene modulations. Finally, we will touch upon the recent studies in which Cpf1 is being utilized for genome editing.

## Evolutionary origins, structural basis, and molecular mechanisms of Cpf1 family proteins

### Evolutionary origins of Cpf1

It is proposed that Cas9 and Cpf1 CRISPR systems have evolved through similar but independent pathways. The search for homologs of the Cas9 and Cpf1 effectors showed that their RuvC-like nuclease domains might be derived from TnpB proteins encoded by autonomous and numerous bacterial and archaeal transposons.

The evolution of Cpf1 CRISPR system started when an IS605 family transposon integrated next to a stand-alone CRISPR array [3]. In this process, functional connection between the CRISPR array and the transposons has evolved to the formation of a polypeptide containing 400 amino acids with loss of mobility. Subsequently, the insertion of additional domains into the transposon happened, which worked as a platform for Cpf1 evolution. Ultimately the Cas1 and Cas2 as essential components for Cpf1 adaptation were recombined to this platform and generate CRISPR–Cpf1 locus [2]. This evolutionary processes resulted in an effector nuclease with unique architecture and functionality.

Upon searching the public sequence databases showing the diversity of Cpf1 family proteins, forty-six non-redundant Cpf1 family proteins have been found, but only 16 Cpf1 candidate proteins were chosen for PAM sequence determination and functional analysis. Among these novel Cpf1 families, only eight members (*Francisella novicida U112*, *Prevotella disiens*, *Acidaminococcus sp. BV3L6*, *Lachnospiraceae bacterium ND2006*, *Lachnospiraceae bacterium MA2020*, *Candidatus Methanoplasma termitum*, *Moraxella bovoculi 237*, and *Porphyromonas crevioricanis*) can induce an efficient cleavage activity on the target DNA with identified PAM sequences [12].

**Fig. 1:**
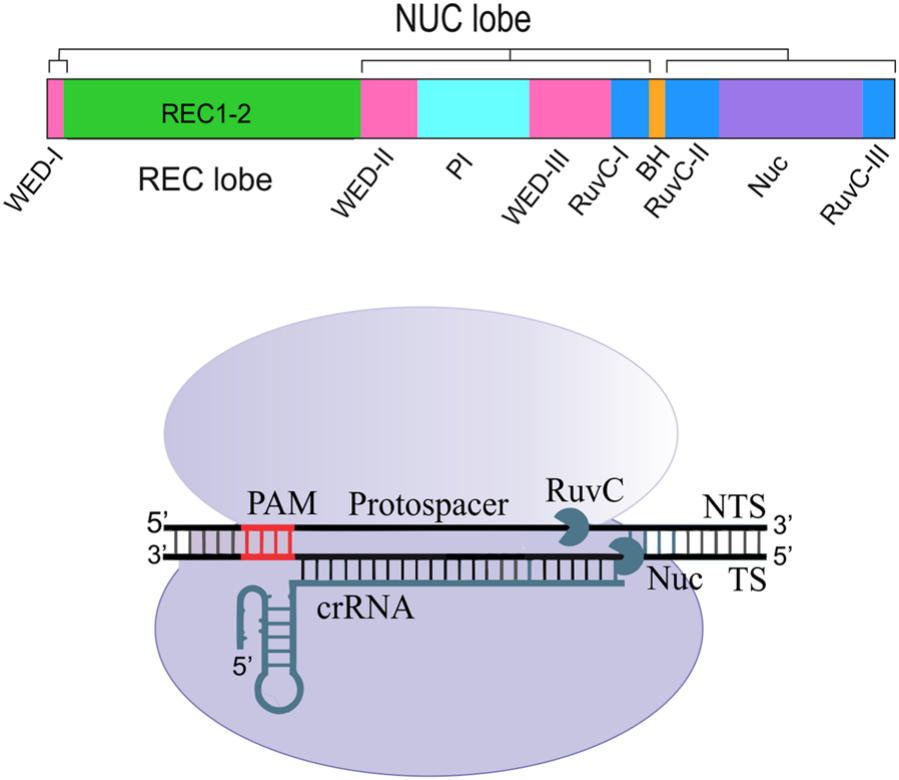
Schematic representation of the Cpf1 domain organization

AsCpf1 from *Acidaminococcus* consists of 1307 amino acids slightly shorter than SpCas9 with 1368 amino acids. The crystal structure of AsCpf1 revealed that it contains two major lobes: a nuclease lobe (NUC) and an alpha-helical recognition lobe (REC) [14, 15]. The REC lobe comprises two domains including REC1 and REC2, while the NUC lobe is composed of the RuvC domain and three additional domains: PI, WED, and BH. The RuvC-like endonuclease domain of Cpf1 is subdivided into three discontinuous segments (RuvC I–III), but it lacks the second HNH endonuclease domain in contrast to Cas9 protein [15] (Fig. 1). Each of these components has a specific responsibility in inducing DSB by Cpf1, which will be touched upon.

Three well-studied orthologs of Cpf1, including AsCpf1, *F. novicida U112* (FnCpf1), and *Lachnospiraceae bacterium ND2006* (LbCpf1), were applied for genome editing in eukaryotic cells [16, 17]. The analysis of uncharacterized Cpf1-family proteins with these three Cpf1 orthologs showed varying degrees of homology. Thereby, Zetsche et al. [18] reported high levels of homology between these orthologs at the direct repeat (DR) sequences of the crRNAs associated with Cpf1. Besides, they suggested the conservation of the mechanism of crRNA maturation within the Cpf1-family due to a strong homology at the stem structure and the AAUU motif, which is required for efficient crRNA maturation [19].

### The biogenesis of Cpf1 crRNA

The CRISPR array of Cpf1 does not need tracrRNA and RNase III for the processing of mature crRNAs, and catalysis of pre-crRNA processing occurs by an intrinsic ribonuclease activity of the Cpf1 itself.

The Fncpf1 CRISPR locus comprises of 27–32 base pair (bp) long spacers located beside 36 bp long repeats and is expressed as a single transcript [12]. Cpf1 directly recognizes the repeat-derived segments in the pre-crRNA transcript that construct the pseudoknot structures [14]. The pseudoknot organizes divalent cations such as Mg^2+^ or Ca^2+^; these cations empower binding of the crRNA to Cpf1 protein [20]. The 5′ end processing of the crRNA is accomplished by a catalytic site in the WED domain, but the mechanism of the 3′ end processing remains unknown. The mature crRNA of Cpf1 consists of 42–44 nucleotides, which contains a 19-nucleotide long repeat and a 23–25 nucleotide long spacer with a short stem-loop structure in the direct repeat sequence [12].

The mature crRNA required for Cpf1 actions is shorter than the guide RNA of Cas9. The shortness of the coding sequence of Cpf1 guide RNA reduces the cost of RNA synthesis and facilitates the delivery of this cassette into target cells. Furthermore, tracrRNA is not required for DNA cleavage in contrast to Cas9, which requires tracrRNA at both steps including the processing of mature crRNA as well as targeted DNA cleavage [21].

### Recognition of the PAM

PAM recognition is the first step in Cpf1-mediated gene editing. When PAM is located in the surrounding of a related protospacer, it will set off subsequent hybridization of the crRNA to the target DNA strand and the formation of an R-loop structure. The PAM sequences of Cpf1 family proteins are predominantly T-rich and differ only in the number of thymidines. Also, it was revealed that the nuclease component of Cpf1 recognizes 5′-TTN-3′ PAM on the target strand [12]. PI, REC1, and WED domains altogether participate in the PAM recognition.

Upon crRNA binding to FnCpf1, PI and WED domains form a cleft, which accommodates the PAM DNA duplex. Two conserved lysines (K613 and K671 in FnCpf1), located in the WED and PI domains, are responsible for PAM readout [15, 20, 22, 23]. Other conserved residues located in PI, REC1, and WED domains bind to PAM nucleotides by formation of Van der Waals and hydrogen bonds. These domains recognize the narrow minor groove of the T-rich PAM DNA at the 5′end in structure- and DNA sequence-dependent manners.

In vitro cleavage experiments demonstrated that although AsCpf1 and LbCpf1 prefer the TTTV PAM (V is A, G, or C), they can also recognize C-containing sequences as suboptimal PAMs [12]. It is clear that the LbCpf1 and AsCpf1 can cleave target sites by recognizing the non-canonical C-containing PAMs (CTTA, TCTA, and TTCA) in mammalian cells. However, the efficiencies of these Cpf1 orthologue with the non-canonical PAM are lower than those with the canonical TTTV PAM [24]. Structural studies have revealed that in comparison to Cas9, the PAM binding channel in Cpf1 is more loosely ordered, leading the channel to be slightly open during suboptimal PAM binding [23]. The lack of specific interactions between the PAM and Cpf1 side chains is an explanation for the lower cleavage efficiencies of Cpf1 on the targets with suboptimal PAMs. Notably, AsCpf1 and LbCpf1 can recognize both these kinds of PAMs in vitro and in vivo.

Recently, the crystal structures of LbCpf1 in complex with the crRNA and its target DNA was investigated. LbCpf1 families, which recognize either TTTA, TCTA, CCCA, or TCCA as a PAM, were analyzed at 2.4- to 2.5-A° resolutions. These structural studies showed that LbCpf1 tolerated conformational alteration to form distinct interactions with the PAM-containing DNA duplex. These conformational changes depended on the PAM sequences, thereby explaining the LbCpf1 PAM preference [23].

Findings showed that their requirement of a TTTV sequence limits the utility of the commonly used AsCpf1 and LbCpf1 as a PAM in the target DNA [12]. Since mutations can change PAM preference of Cas9 in the residues near the PAM DNA duplex [25–27], recent efforts are ongoing to investigate whether the PAM preference of Cpf1 can also be modified. However, different orthologues of Cpf1 have strong sequence and structural homology [12]. In order to obviate the PAM limitation, a structure-guided mutagenesis screen was executed to enhance the targeting range of Cpf1. To this end, Gao and his colleague generated two engineered AsCpf1 variants carrying the mutations S542R/K607R and S542R/K548V/N552R. These variants recognized TYCV and TATV PAMs, respectively, which retained a high DNA-targeting specificity. Note worthily, these innovative variants markedly increased the number of potentially targetable sites of Cpf1 by approximately three fold in human coding sequences to one cleavage site per ~ 11 bp [22].

Although TYCV and TATV PAMs have been efficiently used in human genome editing, findings of Li et al. [28] revealed that between the two modified LbCpf1 variants (RR and RVR), only LbCpf1 (RR) variant enabled efficient editing or multiplex editing of the desired genes containing non-canonical TYCV PAMs in rice. This knowledge expanded the field of LbCpf1-mediated genome editing in rice. It is suggested that the LbCpf1 (RR) variant may be a useful genome-editing tool for both basic research and crop breeding in other plant species. Altogether, we can insinuate that Cpf1 is an appropriate nuclease for targeting the AT-rich genomes such as the genomes of the chloroplast, mitochondria, and *Plasmodium falciparum*.

### Target DNA binding and cleavage

After the recognition of PAM by crRNA-bound Cpf1, binding to the target DNA occurs. This process involves melting of the target dsDNA, which enables base-pairing interaction between crRNA and the target DNA strand. Structural, biochemical, and biophysical studies of Cpf1 have revealed that PAM recognition coincides with DNA strand separation because PAM recognition works as a license for R-loop formation [10]. Similar to Cas9, off-target effect and non-specific cleavage have been documented for Cpf1 [29, 30]. Since sequence specificity is an essential aspect of Cpf1 utilization in therapeutic approaches, this nuclease provides various mechanisms in place that ensure the binding and cleavage of only cognate or near-cognate DNA sequences.

### Initiation of target DNA unwinding

After the recognition of PAM on the target, DNA unwinding and R-loop formation take place. During DNA unwinding, the phosphate group linking the last nucleotide of the PAM and the first target strand protospacer nucleotide interact with some specific amino acid residues. This interaction stabilizes a structural distortion on the target strand [15, 20]. Furthermore, a conserved lysine residue of Cpf1 is inserted in the DNA duplex directly downstream of the PAM promoting DNA unwinding [15]. The structural studies of Cpf1 in its DNA-bound states have revealed that this enzyme makes extensive interactions with the PAM proximal nucleotides on the non-target strand [31]. These findings suggested that the interactions between Cpf1 and PAM sequence contribute to R-loop formation through stabilizing the DNA in the unwound state. It is worth to note that induction of mutations in the specific residues that are involved in binding to the non-target strand, impacts the efficiency of DNA binding and cleavage [32].

### Seed segment binding and RNA–DNA duplex hybridization

The location of seed segment of Cpf1 with 5–6 nt length is at the 5′ end of the crRNA spacer. The seed segment is critical for the initial steps of site-specific crRNA-target strand hybridization [19]. Upon PAM recognition, local unwinding of the target DNA locates the PAM-proximal target strand nucleotides in attachment with the seed segment. Base-pairing interactions with the seed region facilitate crRNA-target strand hybridization and allow the directional unwinding of the DNA and concomitant formation of the crRNA-target strand heteroduplex. Crystal structures of the dual complexes of Cpf1 with its crRNAs have shown that the seed segments of crRNA are preordered in an A-form helical conformation [20]. This preorder prepares the nuclease-crRNA complexes for target recognition by decreasing the entropic penalty associated with the formation of crRNA-target strand duplex. In consequence, it is insinuated that mismatches between the seed region and the target strand DNA have unfavorable effects on DNA cleavage.

More structural investigations showed that the open structure (apo) of LbCpf1 is an elongated form, but changes to a compact form upon binding to crRNA [14]. Similar to LbCpf1, a substantial conformational change from an open to a closed form might be needed for crRNA binding to MbCpf1, while apo FnCpf1 protein adopts a closed conformation. These reports suggested that Cpf1 conformational changes induced by crRNA might be different depending on the species [33].

### DNA cleavage by Cpf1

**Fig. 2:**
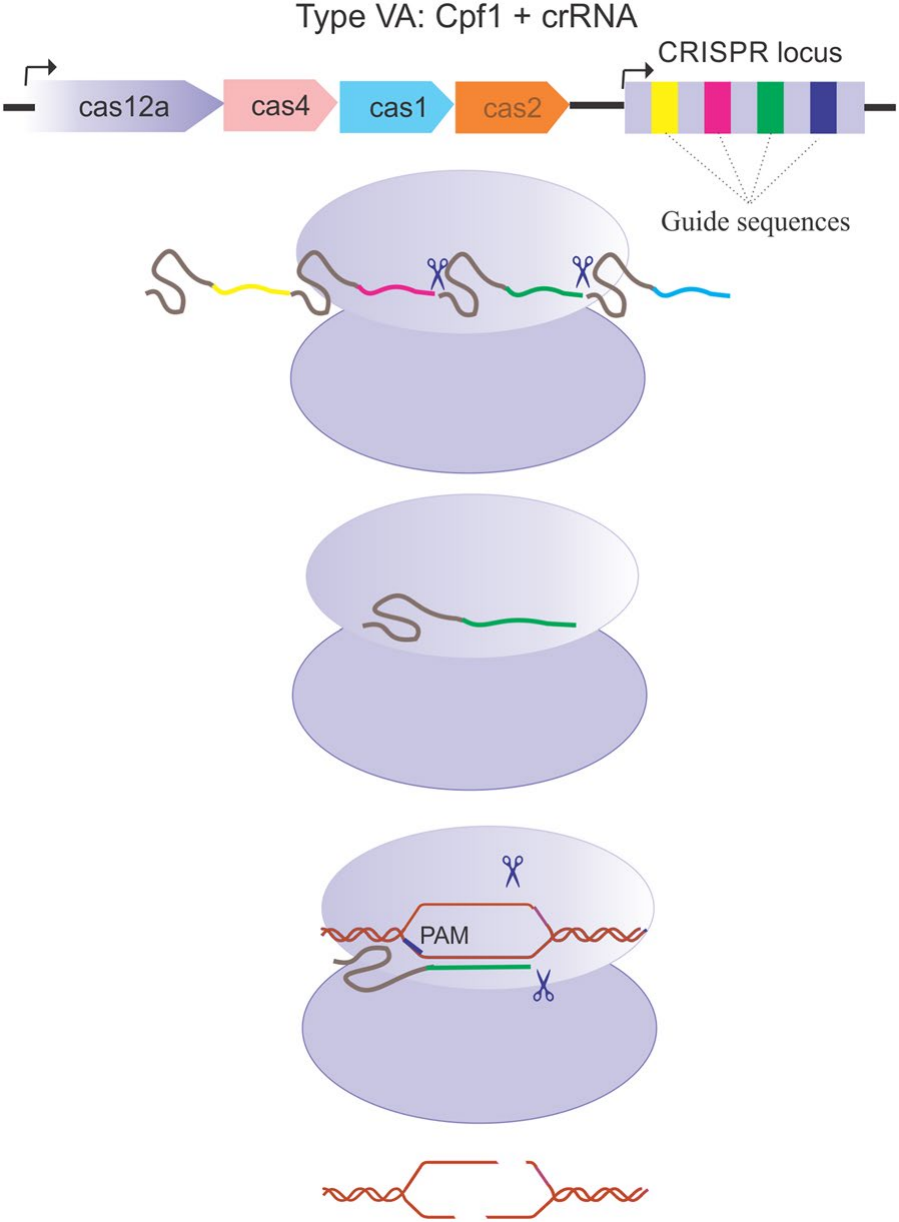
Assembling and activation of CRISPR Cpf1–crRNA complex. Cpf1 (purple) recognizes a pseudoknot that formed by the repeat-derived segment of the pre-crRNA (gray). Subsequent to pre-crRNA binding, Cpf1 itself cleaves the 5′ end of the crRNA by endonucleolytic activity. But, it is currently unclear which RNase processes the crRNA 3′ end. Recognizing a 5′-TTTV-3′ PAM (yellow) in a DNA target (brown) triggers the base pairing of the spacer-derived segment of the crRNA (green) with complementary target DNA. Target DNA cleavage mediated by Cpf1 makes a PAM-distal dsDNA break with 5′ overhangs

Even though the precise mechanism of the DNA cleavage by Cpf1 nucleases remains unclear, the crystal structure of the related Cas12b was applied to resolve this ambiguity [2]. These data beside more recent biochemical insights imply that Cpf1 cleaves both DNA strands through the nuclease activity of a single catalytic site placed in the RuvC domain, while the Nuc domain coordinates the substrate DNA [20]. Moreover, it has recently been revealed that binding of Cpf1 to the target strand activates the RuvC catalytic site. Activated catalytic site triggers the non-specific ssDNase activity, which cuts the target ssDNA in cis and the non-target ssDNA in transpositions [34] (Fig. 2). As the active site of RuvC can only embed one DNA strand at a time, the target and non-target DNA strands are presumably cleaved sequentially [20]. The analysis of the structure of the FnCpf1 pre-cleavage complex implicated that non-target strand might be the first one to be cleaved. This sequential cleavage of DNA elaborates the mechanism of staggered-end DNA break induced by Cpf1. These sticky ends facilitate the integration of the DNA in proper orientation with high accuracy by the NHEJ (non-homologous end joining pathway). NHEJ, which is more frequent than the homologous repair (HR) pathway, remains active throughout the cell cycle; but HR is most active during the S and G2 phases [35]. Conversely, Cas9 makes a blunt end cut at the target DNA and disrupts the gene functions by indel and frame-shift mutations induced by the NHEJ pathway. The usage of the Cpf1 nuclease is now resolving this bottleneck.

In addition, Cpf1 cleaves the target DNA at the distal end of PAM, far away from the seed region. Therefore, the location of indels mediated by Cpf1 will be far from the target site, which is preserved for subsequent rounds of Cpf1 cleavage [12].

## Guide RNA design

The analysis of the structures and biogenesis pathways of crRNAs is imperative for the practical application of genome editing approaches. Programming Cpf1 for genome editing implementation needs the expression or delivery of crRNA alone. The spacer-derived segment of the gRNAs is similar in length in both Cpf1 (~ 24 nt) and Cas9 (~ 20 nt) gRNAs [9]. The gRNA characteristics and target site features are two significant factors that determine the specificity and target efficiency of genome editing mediated by Cpf1. Studying the gRNA attributes related to target efficiency revealed many sequences and structural features of gRNAs such as GC content, minimum free energy, melting temperature, the position-specific nucleotide composition, and the position-non-specific nucleotide composition. Kim et al. [24] used these features to develop an algorithm that predicts target efficiency. A machine learning technology was devised based on the published gRNAs data to forecast the target efficiency for any given gRNAs. CRISPR-DT was the initial web service that assisted users to design optimal gRNAs for the CRISPR-Cpf1 system by acknowledging both specificity and target efficiency [36].

Cpf1-Database is another web-based gRNA design tool, which includes all the available pre-searched targets of Cpf1 with 5′-TTTN-3′ PAM sequences in all coding sequence (CDS) regions within the whole genome of 12 selected organisms. Cpf1-Database provides a rapid and simple way of designing gRNAs for thousands of genes and facilitates the genome-wide screening experiment via usage of Cpf1 [37]. Cpf1-Database is updated frequently by following the recent gene/transcript/CDS annotation information and alternative PAMs of Cpf1 (Table 1).

**Table 1:** CRISPR Cpf1 web base designing tools

Web base tool	Type of Cpf1 orthologues	PAM sequence	Uniform resource locator
Breaking-Cas	AsCpf1	TTTV (in 5′)	http://bioinfogp.cnb.csic.es/tools/breakingcas
	AsCpf1 (RVR variant)	TVTV (in 5′)	
	AsCpf1(RR variant)	TYCV (in 5′)	
Cas-OFFinder	AsCpf1/LbCpf1	TTTN (in 5′)	www.rgenome.net/cas-offinder
	AsCpf1/LbCpf1	TTTV (in 5′)	
	FnCpf1	TTN (in 5′)	
	FnCpf1	KYTV (in 5′)	
CHOPCHOP	Cpf1	TTTN (in 5′)	https://chopchop.cbu.uib.no
CRISPOR	AsCpf1/LbCpf1	TTTV (in 5′)	https://crispor.tefor.net
	AsCpf1/LbCpf1	TTTN (in 5′)	
CCTOP	AsCpf1/LbCpf1	TTTN (in 5′)	https://crispr.cos.uni-heidelberg.de

## On-target specificity and off-target effect

High specificity, as well as low levels of off-target DNA cleavage, are critical elements for genome editing applications of CRISPR-associated nucleases. The seed segment of gRNA and PAM are two elements that determine Cpf1 specificity. It seems that Cpf1 has a lower threshold for non-seed mismatches vis-a-vis Cas9. Although Cpf1 tolerates single non-seed mismatches in vitro, mismatches between any of the first 18 nucleotides in the target DNA and the spacer-derived segment of the crRNA impede the activity of Cpf1 [29, 38]. The suggestion is that protein–DNA interactions stabilize the R-loop structure and facilitate binding of the mismatched off-target DNAs. Hence, the induction of mutation in specific amino acid residues involved in interactions with the target strand or the non-target strand in the R loop would increase the nuclease dependency to guide RNA-target strand DNA base pairing [30]. This strategy may improve the sensitivity to guide RNA–DNA mismatches, which has been exploited to engineer AsCpf1 by mutating non-target strand -coordinating residues [22]. As well as Cas9, the fidelity of Cpf1 variants can potentially be ameliorated by structure-based rational engineering [9].

Other elements that affect Cpf1 activities at the endogenous sites include chromatin accessibility and target sequence composition. Thus, for the prediction of nuclease activity, the chromatin accessibility must be considered. To this end, two algorithms have been proposed to predict the activity of AsCpf1 gRNAs. For designing these algorithms, a deep-learning framework based on a convolutional neural network and chromatin accessibility information were incorporated.

The DeepCpf1 web tool, working based on these algorithms, enables the accurate prediction of AsCpf1 activity in 125 cell lines with available chromatin accessibility information (http://deepcrispr.info) [39].

Recognizing and avoiding off-target effects is a critical step in the application of CRISPR/Cpf1. Off-target effects are related to various factors such as the extent of similarity between the off-target and the sgRNA in the region immediately beyond the seed, the chosen span of the seed chromatin accessibility information, and the methylation status [40]. Hence, improving the efficiency of CRISPR/Cpf1 genome editing and minimizing its off-target effects by using bioinformatics tools has been extensively explored. Recently, CRISPR-offinder has been introduced to predict and validate off-target sites, which may be induced by Cpf1. By using CRISPR-offinder, the cleavage efficiency and CRISPR/Cpf1 off-target effects were assessed [41].

## Cpf1 delivery strategies

Cargo and delivery vehicle are two determinant factors in the cellular transmission of biological agents. Regarding CRISPR system cargoes, there are three commonly utilized strategies: [1] DNA plasmid encoding both the endonuclease protein and the gRNA, [2] endonuclease protein with gRNA (ribonucleoprotein complex: RNP), and [3] mRNA for endonuclease translation besides a separate gRNA [42]. Depending on the type of cargo, the delivery vehicles are determined to pack each of these three cargoes. The other significant issue in choosing delivery vehicles is considering the in vitro as well as in vivo approaches [42].

Vehicles recruited in the transferring of gene editing cargoes are classified into three general categories: physical delivery, viral vectors, and non-viral vectors. The most common physical delivery techniques include microinjection and electroporation. Viral delivery vectors include retrovirus, lentivirus, adeno-associated virus (AAV), and adenovirus (Ad) vectors [43]. Viral vectors are especially appropriate vehicles for in vivo works, most commonly used for CRISPR system delivery. Reported on-viral vector systems include lipid nanoparticles, cell-penetrating peptides (CPPs), and gold nanoparticles.

Among viral vectors, the advantages of AAV and Ad vectors such as the episomal nature, and efficient transduction of DNA into non-transformed, either dividing or non-dividing cells, has led to using these vectors for CRISPR/Cpf1 delivery. The efficiency of AAV vectors carrying AsCpf1 for targeting the primary neuronal cells as well as brain was verified in a mouse model [44].

Among viral vectors, the advantages of AAV and Ad vectors such as the episomal nature, and efficient transduction of DNA into non-transformed, either dividing or non-dividing cells, has led to using these vectors for CRISPR/Cpf1 delivery. The efficiency of AAV vectors carrying AsCpf1 for targeting the primary neuronal cells as well as the brain was verified in a mouse model [44].

Recently, there was a generation of Ad vector-mediated CRISPR/Cpf1 system which proved to be valuable in genome editing in primary human hepatocytes. Despite the lower immunogenicity of AAV vectors than Ad vectors and their more safety for therapeutic applications in humans, AAV vectors have smaller packaging capacity than Ad vectors [43].

Cationic lipids, as versatile carriers, have been applied in a large number of gene editing approaches. In contrast to the efficient delivery of SpCas9 with cationic lipids such as Lipofectamine [45], the efficiency of Cpf1 (RNP) delivery with cationic vehicles is low. This fact may be due to the weak interaction between Lipofectamine and the Cpf1 RNP because the RNP complex of Cpf1 has a low negative charge for interacting with the positive charge of cationic lipids [45].

Cell-penetrating peptides promote the delivery of various molecular cargoes to the living cells through their ability to bind and translocate into cell membranes. However, CPP-mediated protein delivery usually showed a low cytosolic distribution, but the combination of a CPP with endosomal leakage domains (ELDs) improved the endosomal entrapment. CPP-ELDs complex improved the direct delivery of Cpf1 RNP to mammalian cells, including human stem cells, cancer cell models, and hard-to-modify primary natural killer (NK) cells [46]. Also, there was an observation that poly (aspartic acid) derivative polymers could efficiently deliver AsCpf1 to myoblasts in vitro and in vivo [47].

Gold nanoparticles (GNPs) have various applications in applied biomedical science, from imaging to gene delivery. As such, these nanoparticles are wildly utilized in vitro, ex vivo, and in vivo settings. Lately, a conjugation of DNA oligonucleotide-GNPs-Cpf1 RNPs, encapsulated with PAsp (DET) polymer, was used to generate CRISPR–Gold. This innovative construct may significantly promote the brain targeted therapeutics and facilitate the generation of focal brain-knockout animal models [48].

## Strategies to enhance the efficiency of Cpf1

Various strategies are used to enhance the efficiency of Cpf1 endonuclease. These include engineering the crRNA and mRNA as well as using small molecules. Chemical and structural modifications are used to engineer the crRNA and mRNA which would result in an enhanced on-target activity of Cpf1. Application of small molecule to improve the Cpf1 efficiency requires a more profound understanding of the cellular repair systems.

As discussed above, the Cas9-mediated DSB occurs at 3-bp upstream of the PAM site, leading to the targeted sequence modifications via alternative DNA repair pathways including NHEJ or HDR (homology direct repair) [49]. NHEJ induces frameshift and indel mutations, and causes loss-of-function alleles. NHEJ repairs DNA DSB by two pathways including canonical and alternative pathways. In canonical NHEJ pathway, at first, Ku70/Ku80 protein binds to the cleaved DNA end. In the following step, DNA protein kinase catalytic subunit (DNA-PKcs) joins the repair complex, which self-phosphorylates and also phosphorylates other downstream effectors at the repair site [50]. This pathway results in the linking of the DNA ends mediated by DNA ligase IV [51]. In the absence of canonical pathway, the alternative pathway becomes activated, which requires Werner syndrome ATP-dependent helicase [52].

HDR introduces precise point mutations or insertion of a desired sequence at the locus of target. In the HDR pathway, binding of MRN complex (Mre11, Rad50, and Nbs1) to the DSB [50] allows the endonuclease RBBP8 (CtIP) to remove the nucleotides at the 5′-end [51]. Further, nucleotide resections lead to an extension of long 3′ ssDNA overhangs on both sides of the DNA cut site. Replication protein A (RPA) complex coats these 3′ overhangs, which is followed by the generation of a RAD51 nucleoprotein filament. RAD52 catalyzes the replacement of RPA bound to ssDNA with RAD51, and allows annealing of the damaged DNA with a homologous donor DNA. DNA replication via usage of the donor DNA as a template precisely repairs the DNA [53]. Protein kinase ataxia telangiectasia mutated (ATM) is a significant factor in the phosphorylation of at least 12 proteins of HDR repair system [50]. A profound understanding of the involved molecules in the DNA repair seems to be critical in order to enhance the efficiency of genome editing.

CRISPR-mediated gene knockout by NHEJ has worked efficiently (20–60 percent efficiency in mouse embryonic stem cells and zygotes) [54]. However, the precise induction of a single point mutation or HDR mediated knock-in has remained inefficient (0.5–15% efficiency in the targeted nucleotide substitutions using ssDNA donors) [55]. Therefore, efforts continue to enhance the efficiency of the precise CRISPR gene editing as a significant challenge. Traditionally, a small interfering RNA was used to suppress repair proteins such as Ku70/80 and DNA ligase IV, which raised the knock-in efficiency from 5 to 25%. Moreover, the co-expression of adenovirus type-5 proteins (4E1B55K and E4orf6), which degrades DNA ligase IV, also enhanced the gene editing efficiency from 5 to 36% [56]. Also, further investigations showed that small molecules might enhance the Cpf1 efficiency of gene editing which will be discussed in the following paragraphs.

### Increasing the cpf1 efficiency by engineering the crRNA and mRNA

A variety of approaches have been explored to improve the genome editing efficiency and minimize the off-target effects of CRISPR–Cas systems [30, 57, 58]. Chemical and structural modifications of CRISPR–Cas9 resulted in enhancing the on-target activity in a number of human cells. The incorporation of chemically modified nucleotides in gRNAs has been shown to maintain the percentages of indel mutation in CRISPR–Cas9 nuclease system. It was also reported that a chimeric single-guide RNA (sgRNA) with three chemically modified nucleotides at both the 5′ and 3′ ends robustly improved genome editing mediated by Cas9 in human primary T cells. Since the structure of gRNAs plays a critical role in DNA interference for the CRISPR–Cas9 system, Dang et al. [59] showed that extending the duplex and mutating the continuous sequence of thymines increased the knockout efficiency. Successful experiences in the engineering of Cas9 gRNA have created interest in improving the genome editing efficiency by engineering crRNAs and Cpf1 mRNAs.

**Fig. 3:**
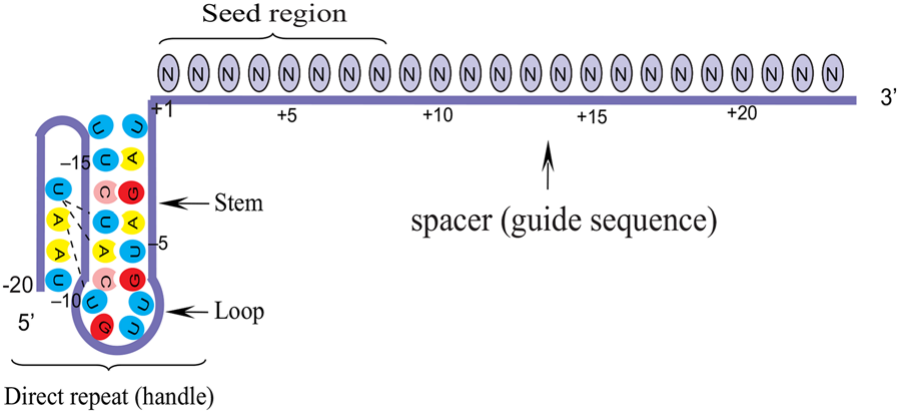
Schematic representation of a mature Cpf1 crRNA. Both the spacer segments and the repeat are derived from the maturation of pre-crRNA

The crRNAs of Cpf1 orthologue in different bacterial species are highly conserved. AsCpf1 crRNA contains a 20-nt direct repeat (DB: known as a 5′ handle) and a 23-nt spacer (guide sequence). DB forms a pseudoknot structure, which is composed of five Watson–Crick base pairs, a noncanonical U–U base pair, one UCUU tetraloop, one reverse Hoogsteen A–U base pair, and three 5′-end bases [15]. The spacer segment, which is complementary to the target DNA, contains a seed region with eight nucleotides length. Seed region flanks at the initial part of the spacer and plays a notable role in the target specificity of CRISPR–Cpf1 system [19] (Fig. 3).

Recently, two strategies including chemical and structural alterations were used to engineer Cpf1 crRNAs. According to the modification pattern applied for Cas9 gRNA, a library of chemically modified crRNA was constructed. The analysis of the crRNA modification on structure–activity relationship indicated the following criteria. Phosphorothioate (PS) modifications usually decreased the genome-editing efficiency. Introduction of five 2′-fluoro modifications at the 3′ end of the handle improved the cleavage activity. The stem duplex in the 5′ handle did not bear splitting, insertion, or deletion of nucleotides. The seed region tolerated small chemical modifications. Concurrent chemical modifications at both the 5′ and 3′ ends of crRNAs were not sufficient. Chemical and structural alterations of AsCpf1 mRNA showed that a ψ-modification was an appropriate chemical alteration for AsCpf1 mRNA.

Furthermore, the combination of guide crRNA and ψ-mRNA substantially enhanced the DNA cutting efficiency up to 300% when compared with the wild-type Cpf1. The application of this combination in the case of LbCpf1 provided a more efficient gene cutting than the AsCpf1. Moreover, it was identified that AsCpf1 and LbCpf1 were different in recognition of crRNA. Notably, LbCpf1 was more conservative in recognition of the loop structure at the 5′ handle compared to AsCpf1. Besides, AsCpf1 showed the ability to recognize the majority of crRNAs from the other Cpf1 orthologue [60].

The extension of crRNA 5′ end enhanced both the editing efficiency and the delivery of AsCpf1 in vitro and in vivo. Additionally, short 5′ extensions improved serum stability via enhancing the tolerance of crRNA 5′ end to chemical modifications. Furthermore, it was revealed that the extension of a 59 nucleotide sequence to the crRNA 5′ end significantly increased the gene editing efficiency both in vitro and in vivo [47].

Broadening the understanding of the AsCpf1 system will facilitate the engineering of the CRISPR–Cpf1 system which as a result will maximize the genome editing efficiency.

### Increasing the Cpf1 efficiency by using small molecules

It has been reported that small molecule compounds can activate or suppress the pathways signaling for DNA repair which may result in the improvement of the CRISPR mediated knock-in efficiency [61]. DNA-PK inhibitors such as NU7026 and NU7441 enhanced the genome-editing efficiency in different cell lines [62]. Also, there was a finding that L755507 and Brefeldin A could amplify the CRISPR-Cas9-mediated genome editing [63]. Besides, Chu et al. and Maruyama et al. asserted that the ligase IV inhibitor SCR7 could also boost the efficiency of genome editing induced by CRISPR-Cas9.

Since Cpf1 is more precise than Cas9, a few studies have investigated the effect of small molecules on the efficiency of Cpf1. In this manner, Riesenberg et al. [62] have utilized a mixture of small molecules, called the “CRISPY” mix. They achieved a 2.3- to 4.0-fold increase in precise genome editing with Cpf1, which has allowed almost 20% of chromosomes to be edited. In another study, the low efficiency of knock-in in hPSCs prompted scientists to use CRISPR-Cpf1 along with small molecules. Chemical screening showed that VE-822 and AZD-7762 enhanced the CRISPR-Cpf1-mediated precise genome engineering in hPSCs. Overall, it can be argued that combination of CRISPR and small molecules, is promising in many applications [64].

## Multiplexed genome editing by CRISPR–Cpf1

The co-expression of a CRISPR-associated nuclease with different gRNAs to simultaneously target multiple genes (multiplexing) is another obstacle to genome editing. The first efforts in the Cas9-based multiplexing were performed in bacteria [65] and mammalian cells [66] by the simultaneous assembly of Cas9 complexes with different sgRNAs (crRNA fused to tracrRNA). In that experience, each sgRNA was transcribed as an individual transcription unit (promoter, gRNA, scaffold, and gene-terminator). Afterward, two methods were found to generate multiple mature gRNAs from a single precursor crRNA, including the utilization of Csy4 (a class 1/type I–F CRISPR-associated RNase) [67] and also tRNA gene positioned between two sgRNA genes [68].

Lack of the need to tracrRNA for specific cleavage of precursor crRNA, has facilitated the use of Cpf1in multiplex genome editing.

Therefore, several methods have been developed to enhance the expression of Cpf1 crRNA via engineering different RNA processing machineries, including Csy4 RNase from a bacterium, self-cleavable ribozyme from a virus [69], and the endogenous tRNA processing enzymes [70]. These RNA processing strategies are applied to generate multiple crRNAs from one primary polycistronic transcript mediated by a Pol II or Pol III promoter.

**Fig. 4:**
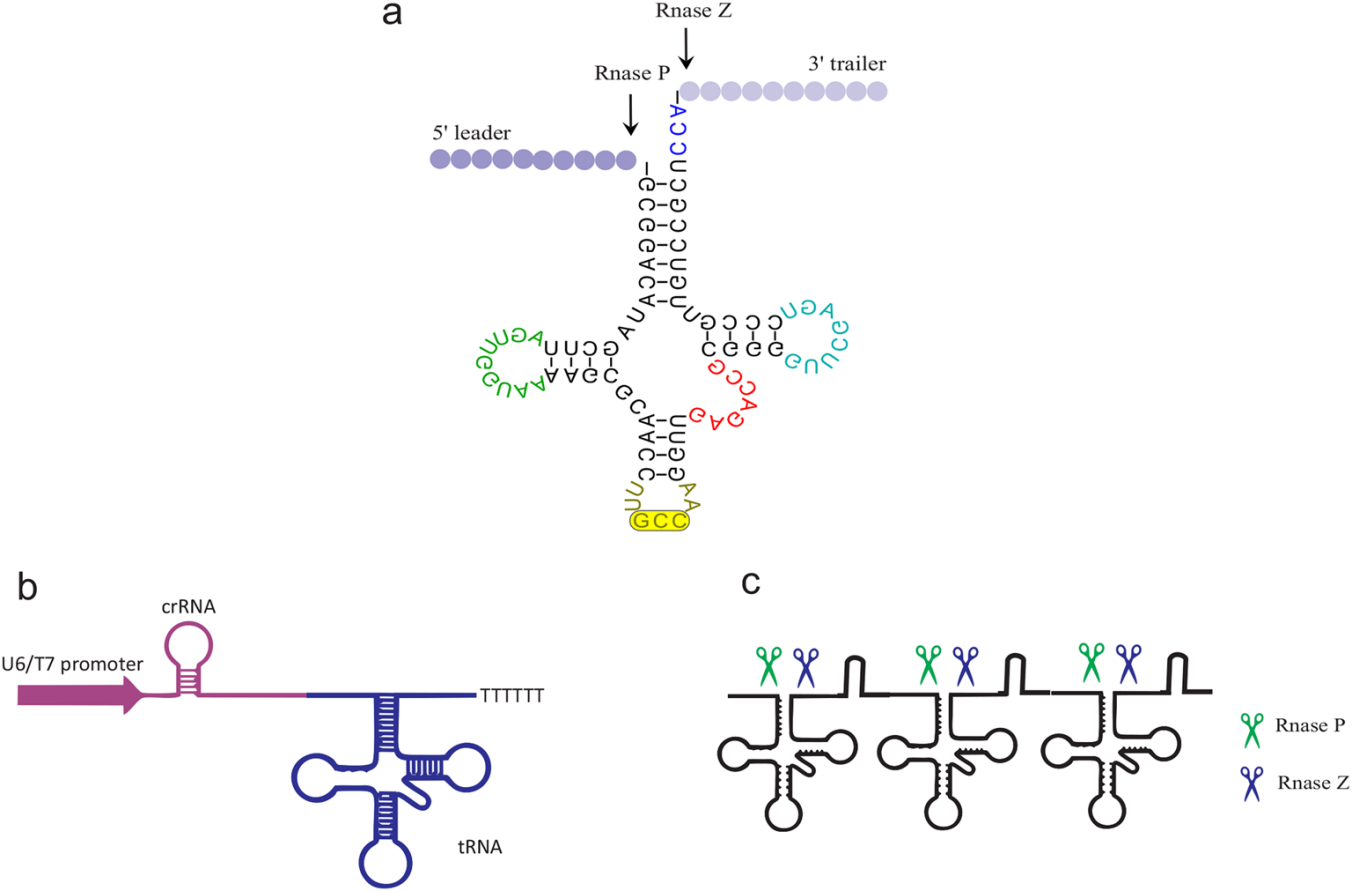
Schematic representation of the Cpf1-gRNA/tRNA expression structure. **a** Secondary cloverleaf structure of the eukaryotic pre-tRNA. In the process of tRNA maturation it can be cleaved by RNase P and RNase Z at specific sites. **b** Structure of Cpf1 gRNA/tRNA system with U6/T7 promoter-gRNA-tRNA. **c** Multiplex gRNA/tRNA system

The catalytic activities of RNA enzymes (ribozymes) are beneficial in the expansion of medical and biotechnological tools. Specifically, the hammerhead ribozyme catalyzed the site-specific cleavage of the phosphodiester bond of an RNA substrate [71]. Therefore, crRNAs ribozyme cassettes, which were driven by different promoters, were utilized in genome editing. To this end, Li and his coworkers have utilized the nuclease activity of ribozymes and have placed the two crRNA ribozyme cassettes in a single array. They named this cassette RCRs (ribozyme-crRNA1-ribozyme–ribozyme-crRNA2-ribozyme). The ribozyme self-catalyzed cleavage resulted in the coordinated expression and release of the designed crRNAs without additional poly U sequences at the 3 terminus [69]. In another effort, the construction of a cassette containing a tRNA gene alternated between two sgRNA genes, resulting in processing of the transcript by endogenous RNase P and RNase Z, and the release of functional sgRNAs in plants [38]. The endogenous tRNA system as a robust reagent can intensify the genome-editing capability and efficiency without requiring additional nucleases or introducing RNAs apart from Cpf1/crRNA (Fig. 4). In addition, intronic PTG (inPTG) uses the endogenous mRNA splicing and tRNA processing machineries to generate gRNAs along with Cpf1. Given the universality of mRNA splicing among eukaryotes, inPTG multiplexing method could be broadly utilized in various eukaryotic organisms to develop CRISPR-based tools [70]. In a recent development, Cpf1-based multiplex genomic editing was successfully introduced into the human cells (HEK 293T), the brain tissue of living mice [44], and yeast [72].

## Modulation of endogenous genes by ddCpf1-mediated technology

In addition to the usage of CRISPR system for genome editing, this technology can be used as a sequence-specific, non-mutagenic gene regulation strategy [73]. The modification of Cas9 nuclease for usage in gene regulation was first implemented by introducing mutations into two nuclease domains of the sp.Cas9 (HNH and RuvC) [74, 75]. These mutations led to a nuclease-deficient Cas9 (dCas9), which is unable to induce DSB but retains the ability to bind to DNA in a sgRNA dependent manner. Hence, dCas9 gives the opportunity for a direct manipulation of the transcription process without the genetically perturbation of the DNA sequence. Also, the combination of diverse effector proteins to dCas9 allowed the gene regulation at the transcription level [76, 77]. Furthermore, it permitted chromosome imaging in live cells and the dissection of long-range chromatin interactions [78, 79].

### ddCpf1-based transcriptional activators

dCas9 and ddCpf1 were applied as transcriptional activators to modify the expression of desired genes by using specific gRNAs that targeted promoters and enhancers. VP64 and p65 are common activator domains that were used as effectors to up-regulate the gene expression in eukaryotic cells [80, 81]. They are readily attached to the C-terminus of both dCas9 and ddCpf1, which resulted in the generation of dCas9/ddCpf1–VP64 or dCas9/ddCpf1–p65. These constructs were able to intensify the expression of the target endogenous genes in human cells [82, 83].

Recently, ddCpf1 from dLbCpf1 was fused with the strong synthetic VPR activator including herpes simplex virus-derived VP16 activator, the Epstein–Barr-virus-derived R trans activator (Rta), and the human NF-KB p65 activation domain [84]. The promoters of three different endogenous genes (HBB, AR, and NPY1R), which are either epigenetically silenced or expressed at low levels in HEK293 cells, were targeted by the dLbCpf1–VPR. The designed crRNAs targeted each promoter flanked at various distances within a distance of 1 kb upstream of the transcription start site (TSS). Findings of this study imply that the fusion of dLbCpf1 and p65 alone exerted little or no transcriptional activation on the target gene promoter, which was consistent with the results achieved by using dCas9. But the drug-regulated ddCpf1-p65 activator (DmrC–p65 fusion with dLbCpf1–DmrA fusions) led to a transcriptional up-regulation. Furthermore, it was demonstrated that the most efficient crRNAs that activated the target gene promoter were positioned between ≈ 600 bp upstream and ≈ 400 bp downstream of the TSSs. These findings thus affirmed the previous discoveries on dCas9 [82].

**Fig. 5:**
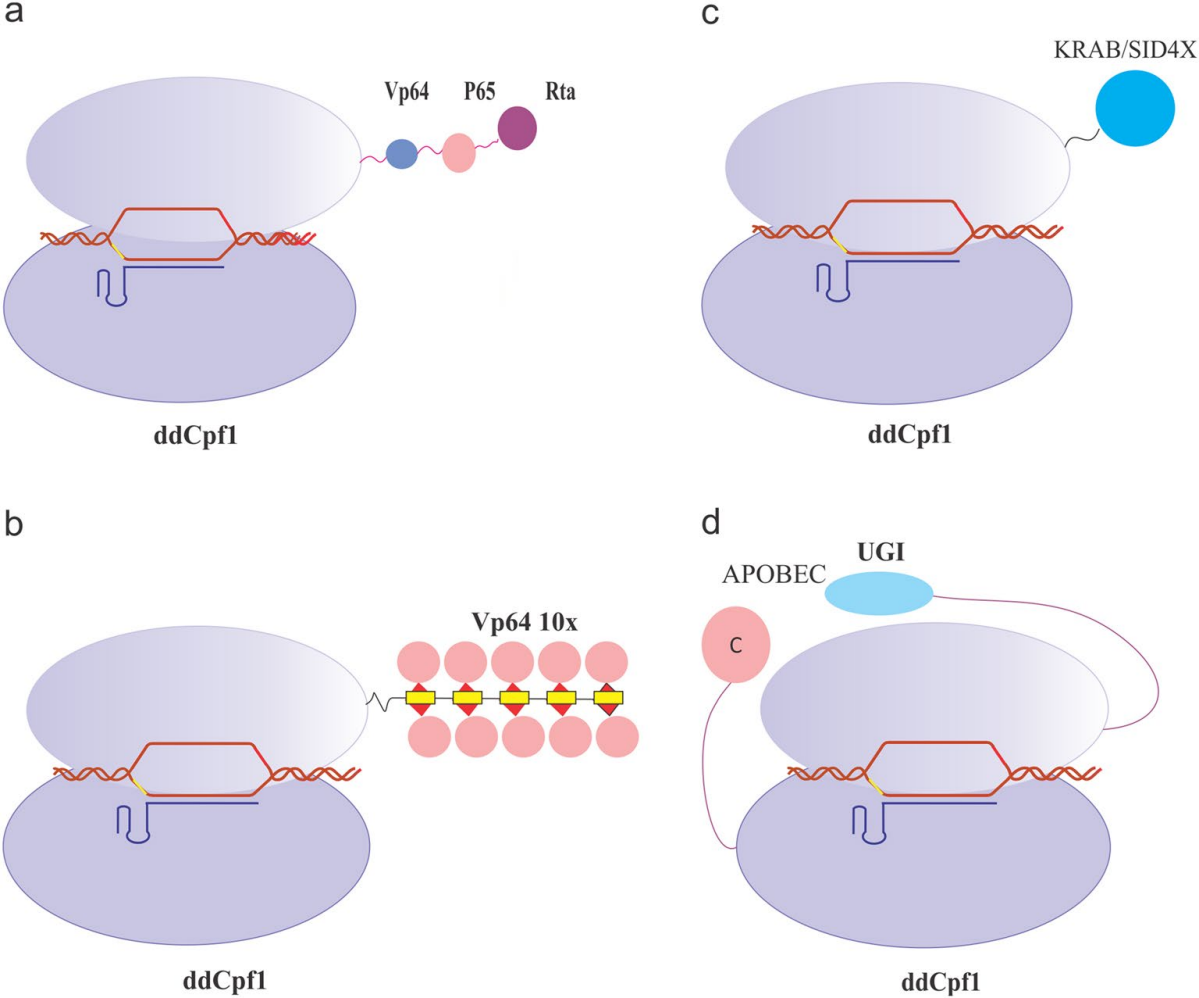
Base editing and control of gene expression by CRISPR-ddCpf1 systems. **a** CRISPR activation (CRISPRa) for gene expression. ddCpf1 can be used as a transcriptional activator by coupling with for instance: four copies of the herpes simplex viral protein 16 (VP16) activation domain (VP64), p65, and the Epstein–Barr virus R transactivator (RTa). This fusion protein called VPR has the ability to augment the gene expression. **b** Sun Tag system is composed of a small peptide epitopes array that recruit multiple copies of single-chain variable fragment (scFv) for fusion with multiple copies of VP64 domains. **c** CRISPR interference (CRISPRi) for gene repression. The CRISPR–CPF1 complex can imped the assembly of RNA polymerases and the promotor in the transcriptional initiation step or perturb the elongation of RNA polymerases to interfere with gene expression. In addition, a transcriptional repressor domains such as Krüppel-associated box (KRAB) can be fused to ddCpf1 to induce gene repression. **d** CRISPR base editing by the complexes of ddCpf1-BE–crRNA–target DNA. This complex is composed of APOBEC1 as a cytosine nucleoside deaminase and DNA glycosylase inhibitor (UGI)

In a different study, dLbCpf1-VPR was tethered to transcriptional activation in 293T cells. The results of the study consistently insinuated that ddCpf1-VPR led to a substantial transcriptional up-regulation of targeted genes. These findings have confirmed that gene activation were more efficient when multiple strong activator domains were fused with ddCpf1, and recruited to the targeted promoter. In addition, the fusion of ddCpf1-VPR demonstrated superior activity vis-à-vis dCpf1-VP64 [85] (Fig. 5a).

The SunTag, synergistic activation mediator (SAM) and VPR systems were the most potent effectors among the activators [86]. SunTag system is a protein-tagging system which consists of multiple scFv-fusing VP64 (where scFv is a single-chain variable fragment antibody against GCN4). Binding of SunTag to the C-terminus of dLbCpf1 (M925) resulted in the efficacious gene activation [87] (Fig. 5b).

### Transcriptional activation by altering epigenetic modifications

The conventional activator domains used as effectors in the transcriptional activation, such as the VP16 tetramer (VP64) [88], work as a scaffold for recruitment of multiple components of the pre-initiation complex [89]. These molecules do not exert a direct enzymatic activity to modify the chromatin state in a specific manner. This method of epigenetic remodeling is not utile in testing the roles which specific epigenetic markers play. It also is not efficient in the direct programming of epigenetic states. Therefore, it is hypothesized that dCas9- acetyltransferase fusion in combination with the target-specific gRNA may change this epigenetic structure which would result in a more effective gene activation [90].

Delvecchio et al. [90] have reported that the histone acetyltransferase (HAT) core domain of human p300 (p300core), comprised of a bromo domain, CH2 region, and HAT domain, maintains intrinsic HAT activity to induce histone H3K27 acetylation at the target loci. Hence, dCas9–p300 fusion protein facilitates the acetylation at the targeted endogenous loci guided by gRNAs, which leads to the regulation of proximal and distal enhancer-regulated genes.

Since CRISPR/Cas9-based acetyltransferase successfully activates the transcription, ongoing efforts are focused on the fusion of dCpf1 with a histone acetyltransferase, which may similarly activate endogenous genes. Zhang and his colleagues prepared the fusion of dLbCpf1-p300core and dAsCpf-p300core and tested their capacity to activate genes. Their findings showed that in the presence of dLbCpf1-p300core, the level of mRNA expression of targeted genes was significantly increased. However, only limited activation was observed in dAsCpf1-p300core transfected cells. Furthermore, promoter based-dLbCpf1-p300core could induce transcription in the cells derived from human tissues other than fetal kidney (HEK239); Accordingly, the cell context was not the determinant factor [87].

As p300 can interact with active enhancers in addition to promoters [91], dLbCpf1-p300core proteins were recruited to target an enhancer about 5 kb upstream of an intended gene. Results showed that dLbCpf1-p300core could activate various genes by targeting enhancers, albeit in a cell context-dependent manner [87].

### dCpf1-mediated transcriptional repression

In addition to gene activation, CRISPR systems possess the ability to function as an RNA interference (RNAi), which has been in use extensively for the silencing of gene expression [92–94]. However, RNAi down-regulates the genes via mRNA degradation in a post-transcriptional level. Observation on dCas9 have hinted that in prokaryotic cells, dCas9 itself functions as an efficient transcriptional repressor. Tightly bound to the DNA, dCas9 sterically hinders the initiation and extension of transcription by interfering with the transcriptional machinery. The gene silencing mediated by dCas9, known as CRISPR interference (CRISPRi) [75, 95], is effectively achieved when the dCas9 is directly guided to a ribosome-binding site or a promoter of the target genes [95]. Furthermore, no difference was reported in the gene repression when dCas9 was bound on either strand, the template or non-template for RNA polymerase, within the prompter [96].

The results of Qi and his co-workers showed a moderate but reproducible knockdown of gene expression (46% repression) when is only Cas9 is used for gene repression within human cells [75]. To induce a robust repression of target genes, it can be suggested to recruit repression effectors such as Kruppel-associated box (KRAB) domain [97], Superman repression domain X (SRDX) and mSin3 interaction domain (SID4X) [98]. These repressor domains may fuse to dCas9 protein (as a fusion protein) [99] or bind to gRNA via aptamers [86]. These silencing systems repress gene expression efficiently in various eukaryotes when upon targeting the proximal region of promoters [100] (Fig. 5c).

In recent years, ddCpf1 has garnered significant attraction for transcriptional modulation especially gene repression. Zhang et al. [101] have stated that dAsCpf1 alone could induce gene repression in Escherichia coli. In contrast to Cas9, the repression efficiency was higher in crRNAs specifically when the target was a template strand of its target genes. Also, in targeting the promoter, ddCpf1/crRNA complex repressed the regulated gene equally well. They also reported that the employment of ddCpf1 in multiplex gene regulation strategy was robust for the quick screening of a library of candidate targets in *E. coli*.

In another CRISPRi study, dFnCpf1 and dCa9-mediated repression were established in the oleaginous yeast Yarrowia lipolytica. The analysis of repression efficiency induced by ddCpf1, dCa9, ddCpf1-KRAB, and dCas9-KRAB indicated no clear correlation between the repression efficiency and the targeting sites. Also, high repression efficiency was observed by using dCpf1 (85%) and dCas9 (92%). Recruiting multiplex gRNAs strategy in this study removed the need to screen effective gRNA loci in advance [77].

ddCpf1 from *Eubacterium eligens* (EedCpf1) is a novel CRISPRi system. This transcription modulator was used to explore the binding strand bias for the CRISPRi system, PAM sequence preference, as well as the tunability of episomal and chromosomal target gene expression. Experimental pieces of evidence demonstrated that EedCpf1 preferred 5′-TTTV-3′ (V = A, G, or C) sequences as a PAM and needed a target binding site on the template strand, within the 5′ UTR or CDS. EedCpf1 induced efficient repression in *E. coli* in both plasmid- or chromosome encoded target gene. This repressions happened when the target was the template strand rather than the non-template strand [102]. Also, dFnCpf1-based CRISPRi system was developed for multiplex gene repression in Streptomyces. Using this novel strategy, a simultaneous repression of three antibiotic biosynthetic genes at efficiencies of ~ 70% was achieved. In the context of dFnCpf1-based gene repression in Streptomyces, crRNA recognized the template strand but not the non-template strand of the target genes, similar to the shreds of evidence observed in *E. coli* [103].

Previously, it has been demonstrated that CRISPR–Cas9 could be engineered to modulate gene expression in plants [104]. The T-rich PAM made CRISPR–Cpf1 a useful reagent for targeting plant AT-rich promoter regions as a transcriptional regulator. To this end, Tang et al. generated dAsCpf1 and dLbCpf1 by inducing mutations in D908A and D832A [12]. Flowingly, they made dCpf1-SRDX fusion proteins as a transcriptional repressor [104]. Under the control of a dual ubiquitin promoter system, the CRISPR–dCpf1–SRDX repressors were transfected into the Arabidopsis. The purpose of these transcriptional repressors was to target the promoter of a noncoding RNA, i.e., miR159b. Both dAsCpf1-SRDX and dLbCpf1–SRDX repressors down-regulated the expression of miR159b less than 10% of the wild-type in randomly chosen T1 transgenic lines. Albeit, a more variation was seen in the case of dLbCpf1–SRDX. These findings suggested that although the nuclease activity of AsCpf1 was less than LbCpf1, it was more tightly bound to DNA [105].

There are only few reports of dCpf1 s function as a transcriptional repressor in mammalian cells. In one of these studies, dAsCpf1–KRAB was constructed and tested in HEK293T cells. Results revealed that dAsCpf1–KRAB in combination with single crRNAs decreased GFP expression, but co-expression of crRNA array with dAsCpf1–KRAB enhanced the GFP inactivation. In addition, the down-regulation of HEK293T endogenous gene (DNMT1) was successful with this approach [106].

Altogether, it is plausible that dCpf1-based CRPISRi system can be vastly applicable to the study of a complex regulatory network of essential genes, as well as a quick screening of functional genes in various species.

## Inducible/split cpf1 variants

The dAsCpf1-based transcription factors provided suitable tools for an efficient gene regulation. Coupling these artificial transcription regulators with signal-responsive modules improves their applications in a gene functional analysis [106].

To extend the utility of dLbCpf1-based activators, drug-regulated versions of these effectors were constructed. Tak and his colleagues used well-characterized DmrA and DmrC domains (fragments of the FK506-binding protein (FKBP) and FKBP-rapamycin-binding protein (FRB), respectively to establish a tunable CRISPRa system. These domains were activated only in the presence of a rapamycin analog called the A/C heterodimerizer. In this approach, split dLbCpf1 activators were fused to a DmrA domain (three or four tandem copies), and a DmrC domain was joined to VPR. In this configuration, the activator was reconstituted only in the presence of A/C drug. The gene activation levels mediated by this strategy were directly correlated with the number of DmrA and VPR domains. However, the maximum activation levels reached were approximately half of that observed with non-split dLbCpf1-VPR fusions [82].

The discovery and application of the recombinase systems such as Cre-loxP, φC31-att, and Flp-FRT enabled a stepwise mutagenesis and genetic events [107]. This system consist of a DNA recombinase (Cre), which specifically recognizes its target DNA (loxP), and catalyzes recombination between two such target sites. By designing a different configuration for the target sites, recombinases have the ability to excise, translocate, and/or invert the targeted DNA. When loxP sites are arranged to point facing each other, Cre recombinase facilitates the inversion of the spacer sequence.

The continuous Cre-mediated inversion would be reversible; therefore mutant loxP sites were generated, which enabled unidirectional Cre inversion. In the recombination process of the mutant loxP sites, lox66 and lox71, this recombination generated a wild-type loxP site and a double-mutant lox72. The affinity of Cre for lox72 was low, hence allowed the floxed DNA segment to invert irreversibly [108, 109]. In a novel fashion, Chow and his co-workers utilized this synthetic Cre-Lox system in sequential mutagenesis in the human cancer cell line. They named this system Cpf1-Flip, which was a lentiviral vector containing LbCpf1, Flip crRNA arrays and the puromycin resistance gene. CrRNA arrays were designed in a way that the first crRNA was encoded on the sense strand, while the second crRNA was inverted. Therefore, using Cre, lox66, and lox71 allowed this system to express the crRNA arrays in the order of their arrangement in the construct [110].

Recruiting FlipArray, it is feasible to engineer more complex CRISPR perturbation programs more readily. These CRISPR systems with two or more crRNAs within an invertible flipArray at their base may provide double knockouts or higher dimensional sequential mutagenesis. In state of the art, utilization of Cpf1-Flip in combination with modified Cre systems such as photoactivatable Cre [111], CreER [112], and split-Cre [113] may lead to even greater control of the FlipArray inversion. Besides, FlipArrays have the potentiality to join with different variants of Cpf1 to be used in sequential and reversible gene activation [83], repression [114], or epigenetic modification [115].

Since dCpf1 is a proper reagent to engineer cellular signaling circuits, riboswitches have been used to modify gene expression controllably. A riboswitch is within the regulatory segment of a messenger RNA molecule, which binds to a small molecule which results in a change in protein production [116, 117]. Insertion of riboswitches into the crRNAs, dAsCpf1-based regulator and the riboswitch-based biosensor prompted the generation of a specific ligand.

The lack of the specific ligand causes the pairing of the crRNA guide region with the antisense stem. Hence, crRNA cannot be bind to its target DNA. But in the presence of the specific ligand, a conformational change induces the crRNA to guide the dAsCpf1-based regulator to the target gene, accordingly regulating gene expression. In this way, Liu et al. integrated a theophylline aptamer into the 3′ end of the crRNAs to target DNMT1. According to their findings, simultaneous addition of theophylline and reprogrammed crRNA–dAsCpf1–KRAB/VPR complex to HEK293T cell culture caused an efficient silencing/activation. This remarks that the crRNA-riboswitch–dAsCpf1 complex modulated the transcription of endogenous genes responding to the external riboswitch-responsive signals causing altered cellular signaling [106].

Multipe chemically inducible systems have been discovered which control the mammalian gene expression. These include Tet on/off system, the nuclear hormone receptor systems, and rapamycin.

Nuclear hormone receptors acting as modular proteins are the reception site for the steroid as well as thyroid hormones. The ERT2 is a truncated form of the estrogen steroid receptor, which binds to the steroid antagonist 4-hydroxytamoxifen but not to the endogenous hormone estrogen.

The inherent properties of ERT2 as a small molecule-inducible system made its usage possible in conjunction with Cre-loxP [118], neurogenic transcription factors [119], Tal effectors [120], and a Zscan4c involved in embryogenesis [121].

Identifying the most proper sites on Cpf1 that can permit the extra amino acids without any conformational interaction is a significant issue concerning the fusion of Cre-ERT2/Cpf1 protein. Monedero et al. [122] showed that Cpf1 is a bi-lobed protein with a “crab claw” shape. The flexible loop around the joint at the top of the “crab claw”, a bump of Cpf1, consists of the amino acids 584 (Glu) and 585 (Lys). This is a favorable location for the Cre-ERT2 to be joined. This is arguably the first report of generating an inducible active Cpf1 with ligand-binding domains of hormone receptors. Their results also revealed that Cpf1 fusion proteins were able to efficiently disrupt both the antibiotic resistant and endogenous gene but at a reduced pace.

## Cpf1 mediated base editing (BE)

Inducing precise single-base changes or base substitutions remains a challenging task. This is due to the inefficiency of HDR in eukaryotic cells [123]. Also, HDR requires the construction of a 500–1000 bp repair template to repair the genomic sequence via the DSB [124]. Localized sequence diversification and single-base substitutions have been in use in a variety of applications from protein functional analysis (especially in basic studies or for gene therapy applications) to treat genetic diseases [125, 126]. Base editing involves site-specific modification of the DNA base through manipulation of the DNA repair machinery to evade faithful repair of the modified base [127]. Base editors are fusion proteins which consist of a site-specific DNA binding module joined to a cytidine or adenine deaminase. Because base editing does not require generating DSBs, this strategy limits the random indel mutations and off-target effects [128]. Various base-editing systems with different architectures, catalytic activities, and potential modifications have been developed. Recently, several groups have been using deaminase-dCas9/Cas9 nickase fusion proteins guided by sgRNA molecules, which resulted in high frequencies of base editing [127]. Cytidine deaminases, such as apolipoprotein B mRNA-editing enzyme, catalytic polypeptide-like (APOBEC), or activation-induced deaminase (AID) and a cytidine deaminase family member, combined to the CRISPR–Cas system led to C-to-T base editing in various species [129].

The properties of base editing are beneficial, and to put it in use, Li et al. [130] generated the first Cpf1 base editor comprised of the fusion of a rat APOBEC1 domain, dLbCpf1, and uracil DNA glycosylase inhibitor (UGI). This base editor, also called dLbCpf1-BE0, promoted effective base editing in sites where Cas9 could not bind, and specifically the ones with a T-rich sequences. dLbCpf1-BE0 induced this editing effect from position 8 to 13 (while the base next to the PAM is counting as position 1) with an efficiency of 20–22%. Moreover, this base editor represented low levels of unintended indel mutations and non-C-to-T substitutions, hence, facilitated the editing of A/T-rich genomic sites (Fig. 5d).

## Differences between Cas9 and Cpf1 in brief

Up to today, the bulk of genome editing via CRISPER has been carries out by using the type II nuclease SpCas9. In the past couple of years, Cpf1 was introduced with various features which SpCas9 lacks. One such feature is the ability of Cpf1 to make staggered cuts whereas Cas9 makes blunt cuts in the genome. Cas9 makes a DSB in target site by cutting each strand in opposite directions. These cleavages result in two double stranded ends. The nuclease active site of Cpf1 cuts target ssDNA in cis and the non-target ssDNA in transposition. This nuclease can only embed one DNA strand at a time, so the target and non-target DNA strands are presumably cleaved sequentially. This sequential cleavage of DNA elucidates the mechanism of staggered-end DNA break induced by Cpf1 [30].

Cas9 needs a complex, hybrid RNA called a tracrRNA (more than 100 nt in length) which functions as a scaffold in the assembly of the CRISPR system. tracrRNA processes and loads the gRNA on the Cas9 proteins. This long, structured RNA can sometimes make difficulty in work with Cas9. Cpf1, on the other hand, does not require any RNA for processing and loading, thus maintaining the functionality with crRNA. This crRNA with a 42-ln nucleotide makes the chemical synthesis of RNA oligos feasible. Cas9 needs a downstream (3′) NGG PAM to bind to DNA, while Cpf1 requires an upstream (5′) TTN. Consequently, TT is slightly more common than GG in the human genome, this minor difference facilitates the search for a suitable site for Cpf1 editing.

Kleinstiver et al. [38] found Cpf1 to be more efficient and highly specific among human cells compared to Cas9, with scarce off-target cleavage capacity. This precision and specificity is due to the inability of Cpf1 to cleave target in the presence of mismatches between any of the first 18 nucleotides in the spacer-derived segment of the crRNA and the target DNA. However, in vitro studies showed that Cpf1 tolerates single non-seed mismatches [20]. In Cas9, on the other hand, nuclease activity has been observed in the presence of up to 4 or 5 mismatches between 20-nt guide sequence and target genome [131]. It is reported that PAM-proximal mismatches are less tolerated when compared with PAM-distal mismatches. Zheng et al. [132] have identified a “core” sequence (4-nucleotides located at + 4 to + 7 position upstream of the PAM) which seems to express enhanced sensitivity to the target-mismatch. These findings demonstrate that Cpf1 is more sensitive to mismatches than Cas9 is which renders Cpf1a plausible candidate for therapeutic applications in future [30].

## Application of genome editing mediated by CRISPR Cpf1

### Cpf1-mediated genome editing in bacteria

The development of CRISPR-Cas9-assisted genome editing technology occurred in order to induce rapid genetic engineering in various bacteria strains. It, although, has faced some limitations such as the toxicity of SpCas9 expression in some industrial strains in addition to the need for complex expression constructs needed for simultaneous targeting of multiple genomic loci [133]. Also, a high-efficient CRISPR-Cpf1 system was used to edit the genome among various bacteria except for the Cpf1 natural hosts.

*Escherichia coli* is known as a versatile organism in biotechnology, and the modifications by various variants of Cpf1 has contributed to this versatility. dAsCpf1-mediated CRISPRi induced specific repression with a high degree of specificity and little off-target effect in *E. coli* [101]. Findings have shown that FnCpf1 and other Cpf1 homologs such as AsCpf1 and LbCpf1 have also interfered with plasmid transformation in *E. coli* [12].

Streptomyces systems of expression produce numerous bioactive natural agents and are utilized as invaluable sources in the realm of drug discovery [134]. The importance of Streptomyces has led to the use of Cpf1 system in genome mining, as well as extraction of novel natural products in addition to the strain improvement. To this end, deployment FnCpf1 in combination with HDR or NHEJ has resulted in efficient gene(s) deletion in *Streptomyces*. Also, a ddCpf1-based integrative CRISPRi platform has orchestrated a single multiplex transcriptional repression of the gene(s). It is note-worthy that FnCpf1 has potentiated efficient genome editing in some significant strains of industrial *Streptomyces* including *S. hygroscopicus* SIPI-KF, which was not editable by SpCas9 [103].

Similarly, the genus *Clostridium* has attracted a large interest; due the potential to produce a large number of chemicals and fuels, such as n-butanol [135]. To increase the yield of butanol produced by *C. tyrobutyricum*, Cpf1-based vectors were used to raise the yield up to 26.2 g/L in a batch fermentation. This enhancement occurred by silencing, and subsequent insertion of two different genes [68].

Furthermore, Cpf1-mediated DNA recombination was achieved in *E. coli*, *Yersinia pestis*, *Mycobacterium smegmatis*, and *Corynebacterium glutamicum* by heterologous expression of FnCpf1, crRNAs, and the addition of ssDNA or dsDNA recombination templates [136]. Note-worthily, FnCpf1 was efficiently tethered for genome editing in Pseudomonas putida KT2440, which proved that CRISPR Cpf1 could be a potent tool to develop the application of *P. putida* KT2440. Also, this was pertinent to the applicability of CRISPR systems in other members of Pseudomonas genus [137]. These studies have also revealed that Cpf1 function might be beneficial in the context of biotechnological applications. Its niche as an efficient genome editing strategy in different bacterial species is a realm for further discovery.

### Cpf1-mediated genome editing in plants

The CRISPR/Cpf1 technology has allowed the plant breeders to ameliorate their yield and quality accurately, and with high efficacy [138, 139]. This use of this system was seen in gene editing within several primitive as well as higher plant species. The green alga *Chlamydomonas reinhardtii*, as a primitive plant, has been the target of Cpf1 RNP co-deliverance with ssDNA repair templates. This in vitro study has resulted in precise and targeted DNA recombination with as much as ~ 10% efficiency. In contrast, employing the same strategy in vivo resulted in extremely low efficiencies concerning the Cpf1 cleavage (0.1–1%), which in principle implied an inefficient gRNA design. Henceforth, it can be argued that the results of in vitro cleavage cannot be applied to the context in vivo studies [140].

Various Cpf1 proteins have been utilized to mediate genome editing among diverse higher plant species such as tobacco, soybean, and rice. Most recently, rice was targeted by Cpf1 to introduce stable and heritable mutations via selection of two genome targets within the OsPDS and OsBEL genes. In another effort, the ability of the FnCpf1 and LbCpf1 to induce targeted gene insertions via HDR and gene silencing via HNEJ were investigated. The evidence obtained from these studies has confirmed the capability of both FnCpf1 and LbCpf1 to generate precise gene insertions as well as indel mutations inside the genome of the rice. Further, the HR-mediated via Cpf1 proved to be more efficient compared to the Cas9 in rice. This efficiency was observed as a dsDNA repair template was provided in conjunction with a Cpf1-enzymes expressing plasmid.

Versatility and easy handling of CRISPR–Cpf1 system permits a wide variety of applications in the genome editing of plants. These applications include functional screening based upon gene knockouts, transcriptional repression, or transcriptional activation, epigenome editing, and the tracking of cell lineages with DNA-barcoding techniques.

To deliver CRISPR/Cpf1, a plasmid expressing Cpf1/crRNA [141] or a complex containing the RNP of Cpf1 and crRNA are introduced into the plant cells [138]. Remarkably, the efficiency of Cpf1-mediated genome editing was higher upon Cpf1 delivery with unprocessed pre-crRNAs compared to the delivery of crRNAs with fully processed 3′ ends [139]. These findings elaborated by in vitro and in vivo assays insinuate a higher binding affinity of Cpf1 for pre-crRNAs [19] which also highlights the importance of crRNA 3′ end for efficient genome editing [38]. Therefore, immature pre-crRNA may improve Cpf1-mediated genome editing in plant cells, but also potentially in other organisms. Also, the lack of the need for tracrRNA presents Cpf1-as an appropriate tool for multiplex genome editing which indicates that Cpf1 is capable of pre-crRNA processing in plants [142]. In conclusion, the broad adoption and simplicity of Cpf1 genome editing technology can augment the plant biotechnology by enhancing the yield as well as the quality of the products.

### Cpf1-mediated genome editing in mammalian cells

In primary cells, NHEJ is deemed as the primary homology-independent repair pathway. Hence, the blunt end DSB mediated by CRISPR/Cas9 systems which are mainly repaired by HDRs turn out to be inefficient in gene insertion when it comes to non-dividing cells [143]. In such bottleneck, the staggered ends induced by Cpf1 might facilitate the homology-independent integration of donor fragments via sticky-ends. On the same token, AsCpf1 and LbCpf1 were successfully recruited to correct the disease-related mutations in patient-derived induced pluripotent stem cells (iPS) along with the germline correction in the mouse model [144]. Also, AsCpf1 was recently shown to successfully perform genome replacement with the donor fragment in primary human hepatocytes prepared from humanized mice with a chimeric liver [43]. Note worthily, AsCpf1-gRNA-tRNA system represented potent targeting efficiency within the mammalian cells such as human cell lines, porcine fetal fibroblasts (PFFs) and also the embryos that include rabbit zygotes and porcine parthenogenetic embryos. Also, this system proved to be successful in generating gene knockout (KO) rabbits with Werner syndrome, dystrophin (DMD) gene-KO pigs, and PLNR14del point mutation pigs [145].

In addition, LbCpf1 had been already in use to generate an APOE knockout rat which can be used as an initial-to-early atherosclerosis model. These APOE knockout rats demonstrated a specific feature of atherosclerosis such as adventitial immune infiltrates. This study hints that the Cpf1 system can manipulate single or multiple genes of rats in vivo [146].

Comparing the genome editing efficiency and accuracy of Cas9 and Cpf1 homologs in various mammalian cell lines (HEK293 and mouse N2a cells), there was an equal or slightly lower efficiency of AsCpf1 and LbCpf1 albeit with a more accuracy compared to SpCas9 [147]. The efficiency analysis of four other Cpf1 orthologes including ArCpf1, BsCpf1, HkCpf1, and PrCpf1 in human and mouse cell lines revealed the inferior efficiency of these orthologues in comparison with SpCas9 [148].

In another study, the ability of engineered FnCpf1 was analyzed for genome editing in HEK293. Findings showed that FnCpf1 with 21 nt spacer sequence had better activity and higher fidelity. Furthermore, FnCpf1 prefers T and G nucleotides for the fourth position of the PAM (5 -NTTN-3) [149].

Recently, CRISPR-based genome editing of the brain has shown great potential in the treatment of fragile X syndrome (FXS). The localized editing will potentially be targeting a brain gene by using local intracranial injection. Lately, the Fmr1 knockout mice, generated by both Cas9 and Cpf1, has been observed to exhibit alleviation in exaggerated and repetitive behaviors which are the hallmarks of FXS [48]. Previously, the efficiency of Cas9 to correct the disease-causing mutations was confirmed in Duchenne muscular dystrophy (DMD) in mouse models [150]. On par with Cas9, it is imaginable that Cpf1 has also the capacity to modify muscular dystrophy in both human and mice cardiomyocytes [144]. Considering the high specificity of AsCpf1 and LbCpf1 compared to Cas9, Cpf1 proteins have a high potential in gene editing, therapeutically speaking.

### Cpf1-mediated genome editing in other organisms

AsCpf1a and LbCpf1a have shown remarkable value in genome editing in vertebrate, including mice, porcine (female embryos), frogs (Xenopus), and zebrafish [17, 151]. AsCpf1a induced DSB in mouse with 50% efficiency, while the efficiency of this Cpf1 orthologue was lower in pigs by 20%. AsCpf1a was unable to efficiently edit the zebrafish and xenopus genome due to being temperature sensitive [151]. However, LbCpf1 allowed an efficient mutagenesis in zebrafish and Xenopus. These findings exhibited the temperature-modulated nature of activity for Cpf1 that controls its ability to access genomic DNA, which was stronger for AsCpf1 [17].

A recent study has demonstrated efficient genome editing with AsCpf1 in Drosophila melanogaster by direct plasmid injection into the embryos or also by genomic integration of Cpf1 and crRNAs [152]. However, the efficiency of genome editing by AsCpf1 was seen to be lower than SpCas9. *Bombyx mori*, an insect species, was another target of AsCpf1. In this case, results showed that the efficiency of AsCpf1 was equal to SpCas9 and SaCas9 when CRISPR constructs were delivered via transfection or microinjection [153].

AsCpf1, FnCpf1, and LbCpf1 were used for genome editing in the model yeast, *Saccharomyces cerevisiae* [72, 154]. The genome editing efficiencies of FnCpf1 and LbCpf1 were comparable to Cas9, while the efficiency of AsCpf1 was lower [154]. The findings of Li et al. also demonstrated that FnCpf1 targeted singleplex, doubleplex, and tripleplex genomic integration of the in vivo assembled DNA parts with efficiencies of 95, 52, and 43%, respectively [155].

Altogether, CRISPR Cpf1 system exerts promising genome editing effects in vertebrates, insects, and yeasts. However, the efficiency of each orthologue is different in the same organism.

### Nucleic acid detection mediated by Cpf1

Today, time and cost-effective nucleic acid detection methods are in high consideration in human genotyping and pathogen detection. Recently, Chen and colleagues reported the application of CRISPR technology in the diagnosis of invading nucleic acid. CRISPR-Cpf1, however, can exert its capability over single-stranded DNA substrates. It also cuts double-stranded DNA sequences in a crRNA-dependent manner. Although recent investigators have observed that Cpf1 also cleaves other nonspecific single-stranded DNA molecules, this non-selective nucleic acid cleavage is triggered only when the crRNA binds to its complementary target, thus allowing Cpf1 to attack other invading nucleic acids in the vicinity by collateral damaging [34]. This evidence provided the platform for a diagnostic tool called DNA endonuclease-targeted CRISPR trans reporter (DETECTR). DETECTR depends on the collateral cleavage of a reporter nucleic acid that is comprised of a single-stranded DNA bearing a fluorophore and a quencher attached to either end. Since the reporter DNA remains unscathed, fluorescence is quenched, and when the collateral cleavage leads to degradation of the intervening DNA by Cpf1, the fluorophore is separated from the quencher, and causes the emission of a fluorescent signal. To enhance the sensitivity of detection, DETECTR also includes a pre-amplification step. In order to achieve higher analytical sensitivity, an isothermal enzymatic reaction is recruited to replicate the nucleic acids in the unknown sample. Note worthily, this technique is rapid and can be established on the lateral flow without any need for specialized equipment [34]. A one-hour low-cost multipurpose highly efficient system (HOLMES) is a promising example of Cpf1 detector platform for high-paced detection of the target DNA. If the target DNA exists in the reaction sample, Cpf1/crRNA of the HOLMES forms a ternary complex with the target DNA and triggers the trans-cleavage of non-targeted ssDNA. Degradation of reporter DNA leads to illumination of the HEX, N12, BHQ1 or other fluorescents.

To find the most efficient Cpf1 for HOLMES, Li et al. [156] assessed ten Cpf1 variants. Their results elucidated that Lb Cpf1 has excellent performance with high signal-to-noise ratios.

To improve the sensitivity, HOLMES was combined with PCR that resulted in an increase in sensitivity from 0.1 nM to 10 aM. Besides, they found that shorter guide sequences used in HOLMES might augment the fluorescence signals up to two-fold. By the fact that there might be no proper PAM sequence in the 5′ site of SNP, Li et al. [156] have designed unique PCR primers which can introduce the PAM sequence.

Promising properties of HOLMES open a new window for its usage as a diagnostic tool in human maladies. Findings have affirmed that HOLMES can determine both homozygous and heterozygous genotypes with high specificity. Furthermore, HOLMES could also be used to detect DNA viruses and RNA viruses such as pseudorabies virus (PRV) and Japanese encephalitis virus (JEV) with a sensitivity as low as 1–10 aM. Rapid detection of nucleic acids by HOLMES provide an opportunity to use this detector in a variety of applications including food as well as environmental monitoring [156].

## Conclusion

Numerous applications of CRISPR Cpf1 in genome editing among various organisms such as bacteria, plant, insect, and mammalian cells have paved the way its usage as an alternative to Cas9. However, these CRISPR systems share significant molecular features, and occupy the same niche in prokaryotic immune systems, albeit with a distinct evolutionary lineage. Structural differences between Cpf1 and Cas9 result in diverse mechanisms of action in the crRNA processing, DNA binding, and DNA cleavage. Even though Cpf1 provides rigorous nuclease activity, the outcome depends critically on environmental factors including the cell type, target gene sequence, and the epigenetic state of the chromosome. T Cpf1 lacks the need for tracrRNA in crRNA processing, and this makes it a surpassing subject in therapeutic approaches. These promising feature of Cpf1 renders the multiplex targeting feasible and increases the coefficient of coincidence in the target locus. Also, collateral cleavage activity facilitates the application of this nuclease on lateral flow which is cost-beneficial in nucleic acid detection. Efforts are ongoing to understand more and more about Cpf1 structure and mechanisms of action. Such knowledge opens a new window for the engineering of this nuclease. This new reality can lay the ground to achieve highly efficient genomic toolkits for genome editing, and to further sophisticate diagnostic approaches.

## Abbreviations

CRISPR: clustered regularly interspaced palindromic repeats
Cas: CRISPR associated proteins
Cpf1: CRISPR from *Prevotella and Francisella1*
spCas9: *Streptococcus pyogenes* Cas9
PAM: protospacer adjacent motif
tracrRNA: Trans-acting crRNA
Fn Cpf1: *Francisella novicida* Cpf1
As Cpf1: *Acidaminococcus* Cpf1
LbCpf1: *Lachnospiraceae bacterium* Cpf1
Mb Cpf1: *Moraxella bovoculi* Cpf1
Pc Cpf1: *Porphyromonas crevioricanis* Cpf1
NUC: nuclease lobe
CPPs: cell-penetrating peptides
AAV: adeno-associated virus
Ad: adenovirus
RNP: ribonucleoprotein complex
ELDs: endosomal leakage domains
NK: natural killer
GNPs: gold nanoparticles
NHEJ: non-homologous end joining pathway
HDR: homology direct repair
dCas9: deficient Cas9
ddCpf1: DNAase deactivated Cpf1
VP64: VP16 tetramer
HAT: histone acetyltransferase
RNAi: RNA interference
CRISPRa: CRISPR activation
CRISPRi: CRISPR interference
APOBEC: catalytic polypeptide-like
AID: activation-induced deaminase
FXS: fragile X syndrome
HOLMES: one-hour low-cost multipurpose highly efficient system
